# Polylactide (PLA) and Its Blends with Poly(butylene succinate) (PBS): A Brief Review

**DOI:** 10.3390/polym11071193

**Published:** 2019-07-17

**Authors:** Shen Su, Rodion Kopitzky, Sengül Tolga, Stephan Kabasci

**Affiliations:** 1 Department of Bio-based Plastics, Fraunhofer UMSICHT, Fraunhofer Institute for Environmental, Safety, and Energy Technology, Osterfelder Str. 3, 46047 Oberhausen, Germany; 2 Department of Mechanical Engineering, Ruhr-University Bochum, Universitaetsstr. 150, 44780 Bochum, Germany

**Keywords:** polylactide, poly(butylene succinate), PLA/PBS blend, compatibility

## Abstract

Polylactide (PLA), poly(butylene succinate) (PBS) and blends thereof have been researched in the last two decades due to their commercial availability and the upcoming requirements for using bio-based chemical building blocks. Blends consisting of PLA and PBS offer specific material properties. However, their thermodynamically favored biphasic composition often restricts their applications. Many approaches have been taken to achieve better compatibility for tailored and improved material properties. This review focuses on the modification of PLA/PBS blends in the timeframe from 2007 to early 2019. Firstly, neat polymers of PLA and PBS are introduced in respect of their origin, their chemical structure, thermal and mechanical properties. Secondly, recent studies for improving blend properties are reviewed mainly under the focus of the toughness modification using methods including simple blending, plasticization, reactive compatibilization, and copolymerization. Thirdly, we follow up by reviewing the effect of PBS addition, stereocomplexation, nucleation, and processing parameters on the crystallization of PLA. Next, the biodegradation and disintegration of PLA/PBS blends are summarized regarding the European and International Standards, influencing factors, and degradation mechanisms. Furthermore, the recycling and application potential of the blends are outlined.

## 1. Introduction

In recent years, the need for a change in the raw material economy has become significant, due to increasing concerns about limited fossil resources and environmental issues. Based on sustainable United Nations development goals [1] “climate protection, environment, resource efficiency and raw materials” are addressed as core issues of “Horizon Europe” by Fraunhofer-Gesellschaft and its partners in the European Research and Technology Organizations (RTO) [2]. A sustainable, circular plastics economy is promoted by using alternative bio-based feedstock for producing plastics and biodegradable plastics as a specialized solution in a circular plastics system [3].

Polylactide (PLA), also known as poly(lactic acid) is a polymer derived from renewable resources. Presently it is produced, besides some smaller plants, in two world-scale plants by NatureWorks LLC in the USA and Total Corbion in Thailand. The predicted PLA production capacity will increase significantly from 2018 to 2023 [4]. PLA is a versatile polyester, but in several applications, the major weakness, its brittleness, has to, be overcome. Therefore PLA is often blended with a ductile polymer such as polycaprolactone (PCL) [5], polyethylene glycol (PEG) [6], poly(butylene adipate-co-terephthalate) (PBAT) or poly(butylene succinate) (PBS) [7,8]. Among these, PBS is a commercially available bio-based and biodegradable polymer [9,10] produced mainly by Showa Denko and Mitsubishi Chemical Corporation with a production capacity of around 100.000 tons per year [4]. Compared with other biopolymers, PBS has a better eco-efficiency, depending on End-of-Life (EOL) options [11].

In the last two decades, a lot of investigation has been carried out with the aim of improving the toughness and crystallization of PLA using PBS. Furthermore, some studies have focused on the degradation behavior of PLA/PBS blends.

However, to the best knowledge of the authors, no comprehensive review has been written covering the research on the modification and degradation of PLA/PBS blends. That is the reason for composing a systematic summary in the present paper.

## 2. Structure and Properties of Neat Polymer PLA and PBS

This chapter summarizes the chemical structure and properties of the two polymers: PLA and PBS.

### 2.1. Building Blocks and Synthesis

**Figure 1:**
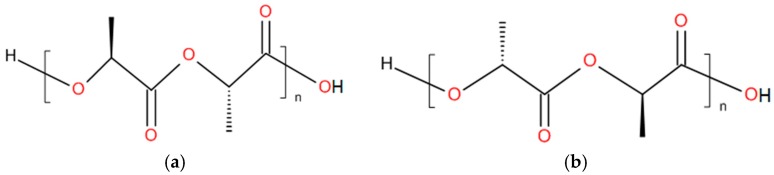
Chemical structure of (**a**) PLLA and (**b**) PDLA.

The building block of PLA is either lactic acid or lactide. The ring-opening polymerization (ROP) of lactide is used to synthesize PLA with high molecular weights (>10^5^ g/mole) [12]. Polylactide possesses two optical active and crystallizable isomeric forms: PLLA and PDLA (Figure 1). Between both, a stereocomplex can be formed.

**Figure 2:**
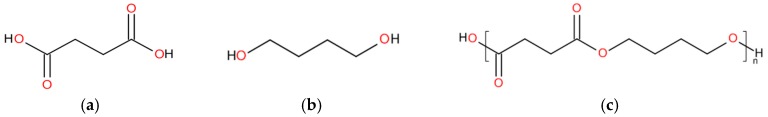
Chemical structure of (**a**) succinate acid, (**b**) 1,4-butanediol, and (**c**) poly(butylene succinate) (PBS).

Succinic acid and 1,4-butanediol are building blocks for synthesizing PBS (Figure 2) [10]. BioAmber Inc. started the commercialization of bio-based succinic acid in 2010. Novamont S.p.A. began producing 1,4-butanediol from renewable resources in 2016 [13].

### 2.2. Polymer Properties

The thermal and mechanical properties of PLA and PBS are given in Table 1. Statistically arranged poly(d,l-lactide) (PDLLA) exists in addition to pure stereoisomers of PLA, the poly(L-lactide) (PLLA) and the poly(d-lactide) (PDLA). While PDLLA is usually amorphous as it has no chain regularity for crystallization, neat PLLA and neat PDLA are semi-crystalline [14]. By blending PLLA and PDLA, a stereocomplex-PLA can be formed [14,15] having a melting point that is approximately 50 °C higher than that of each homopolymer [15]. In general, PLA has many favorable features such as high modulus of elasticity, high strength, high transparency (in the amorphous state) and easy processing [16,17]. However, PLA has disadvantages such as high brittleness, low heat distortion temperature and slow crystallization rate, which can be economically crucial in several applications. PBS also has many desirable properties such as high flexibility and excellent thermal stability. However, its stiffness and melt viscosity for processing are often insufficient for various end-use applications [18].

In addition to the good availability and attractive performance of PLA and PBS and the associated potential for substituting conventional plastics, their biodegradability can be important for several applications. For details, see the chapter “Degradation”. Nevertheless, the high price, especially of PBS, is a hurdle for some applications.

The following chapters of this review summarize the strategies to overcome the limitations of PLA by introducing biopolymers with PBS.

## 3. Toughness Modification

In this review, the modification is subdivided into the following topics: Simple blending, plasticization, reactive compatibilization. and copolymerization. Table 2 gives an overview of literature results for toughness modification by order of the methods, ratios of PLA/PBS, and modifiers.

### 3.1. Simple Blending

The simple blending of PLA with PBS is a practical and economical method to achieve a combination of the desirable properties of each component [7,22,23] and to form a potential matrix for preparing composites without losing their biodegradable behavior [7,24].

The properties of polymer blends depend strongly on the miscibility (or immiscibility) of the polymers. To our best knowledge, no thermodynamic based study concerning the miscibility issues of PLA and PBS have been published besides the papers of Park et al. [25,26]. Based on melting point depression measurements, they published χ-interaction parameters of −0.15 and 0.011 indicating miscibility in the melt. Contradictory to this, other researchers mentioned immiscibility in the molten state [22,27]. In this review, miscibility is a thermodynamic term describing the behavior of a polymer blend by specifying the number of phases [28]. In comparison, compatibility is a technical term defining the property profile of the blend in view of a certain application [28]. To estimate miscibility or compatibility, often the concept of Hansen´s three-dimensional solubility parameters is used [29]. Hansen solubility parameters for PLA are: Dispersion component 18.6 J^0.5^ cm^−1.5^, polar component 9.9 J^0.5^ cm^−1.5^, and hydrogen-bonding component 6.0 J^0.5^ cm^−1.5^ [30,31]. However, values for PBS could not be found. An estimation can be done with group contribution methods, for example, based on three-dimensional data [32] as reported by Valerio et al. [33]. The difference in the parameters of 1.2 J^0.5^ cm^−1.5^ resulting from this approach is much higher than the value calculated with the one-dimensional Hildebrandt solubility parameter of 0.37 and 0.23 J^0.5^ cm^−1.5^ in the glassy and rubbery state, respectively, based on group contribution data from the book of Robenson [34]. This may indicate the sensitivity of the PLA/PBS system to polar group constitution components. 

**Figure 3:**
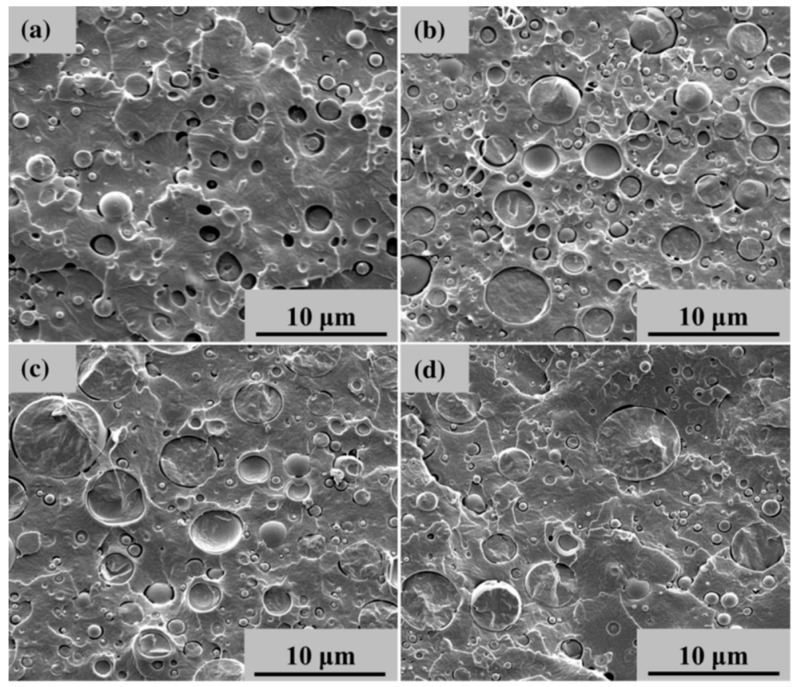
SEM images of PLA/PBS blends at different PBS contents (**a**) 10 wt%, (**b**) 20 wt%, (**c**) 30 wt%, and (**d**) 40 wt% with particle diameters from 0.2 to 6.5 µm [35]. Reprinted by permission from Springer Nature: Polymer Bulletin, Accelerated hydrolytic degradation of poly(lactic acid) achieved by adding poly(butylene succinate), Wang, Y.-P.; Xiao, Y.-J.; Duan, J.; Yang, J.-H.; Wang, Y.; Zhang, C.-L., Copyright © Springer-Verlag Berlin Heidelberg 2015.

The theoretical immiscibility of PLA and PBS matches the observation of only slightly depressed glass transition temperature of PLA in DSC measurements of PLA/PBS blends [16,24] and the biphasic morphology of PLA/PBS blends in SEM micrographs (Figure 3) [35]. Despite the thermo-dynamic immiscibility of the two components, PLA and PBS have some compatibility between each other [16] due to miscible low molecular weight parts of polymers.

PLA/PBS blends can be prepared by solution blending [36,37] or melt blending [7,9,22,38,39]. The solution blending method usually includes the dissolution of polymer blend components in a suitable solvent, mechanical mixing, and solvent evaporation [12]. The type of solvent and its evaporation conditions may affect the morphology of the precipitated phases. By using the melt blending method, polymer blend components are mixed together at a temperature above their melting temperature [12]. In many studies, PLA is used as a polymer matrix while PBS acts as the dispersed component in the blend. When PBS is the minor component, the blend exhibits increased elongation at break and decreased tensile strength and modulus [38]. When PLLA is the minor component, the blend is toughened by stiff PLA particles [38].

The preparation method and blending conditions have a great influence on the material properties. Zhang et al. [38] reported that the elongation at break has a distinct dependence on the blend composition, whereas Liu et al. [39] and Bhatia et al. [9] did not show this relation. Therefore, it is important to pay special attention to the differences in the manufacturing process of the blends and the preparation of test specimens.

### 3.2. Plasticization

Plasticization is another way to improve the ductility of PLA-based materials. The use of a plasticizer reduces the intermolecular forces and improves the mobility of the polymer chains, thereby improving processability and flexibility [40]. However, plasticizers must be chosen with respect to the solubility concept [31], otherwise, they may exhibit poor miscibility with PLA or PBS [28,41].

Based on the plasticization of PLA using oligomeric poly(ethylene glycol) (PEG *M_w_* = 4000), Pivsa-Art et al. reported the effect of PEG on PLA/PBS blends. Adding PEG to PLA/PBS blends (90/10, 80/20, 70/30, 60/40) results in a significant decrease in the glass transition temperature of PLA and a slightly decreased glass transition temperature of PBS. The crystallization rate of PLA is lowered. With increasing PEG content from 0 to 10 phr, the tensile modulus and the strength decreases, fitting roughly the rule of mixture, while the elongation at break slightly improves. Furthermore, the Izod impact strength significantly increases with the addition of 5–6 percent PEG to PLA/PBS (80/20, 70/30, or 60/40) blends [36].

By adding isosorbide diester (15 wt%), a bio-based plasticizer, the PLA/PBS (80/20) blend shows a more homogeneous morphology, a drastically enhanced elongation at break (~250%), and a lower cold crystallization temperature (~78 °C) than unmodified PLA/PBS (80/20) blends (elongation at break: 10.5%; cold crystallization temperature: ~107°) [41].

### 3.3. Reactive Compatibilization

Reactive compatibilization is considered to be a method for suppressing the phase separation and improving the compatibility of immiscible polymer blends [42,43,44]. In the mixing process, reactive compatibilizers react with both blend components to couple the phases within a short processing time [28], thereby enhancing the compatibility and interfacial interactions of the blends [45]. They must be distributed at a high rate in the polymer melt during blending [28]. A large number of reactive compatibilizers for PLA/PBS blends have been reported, for example diphenyl diisocyanate (MDI) [46], lysine triisocyanate (LTI) [42], lysine diisocyanate (LDI) [42], glycidyl methacrylate (GMA) [47], dicumyl peroxide (DCP) [16,44,48], benzoyl peroxide (BPO) [43], organoclays and epoxy functionality-containing components [49], epoxy functionality-containing components (Joncryl^TM^) [24].

MDI plays a role as a reactive coupling agent in PLA/PBS blends containing terminal hydroxyl groups, where urethane linkages are formed [46]. By adding MDI (0.5 phr), the PLA/PBS (70/30) blend exhibits a significant improvement on the elongation at break from 25% to 285%, while the elongation at break of unmodified PLA is 0.5% [46].

The presence of LTI (0.15 phr) and LDI (0.5 phr) results in an enhancement of ultimate strain (more than 150%) of PLA/PBS (90/10) blends [42]. An increase of particle number and decrease of particle size have been detected by laser scanning confocal microscope (LSCM) [42].

The incorporation of GMA (10 phr) improves the elongation at break by over 4000 times in a PLA/PBS (50/50) blend [47]. Meanwhile, the crystallinity is lowered as a result of the reaction between GMA and PLA molecules which increases the difficulty for the orientation of PLA molecules [47].

**Figure 4:**
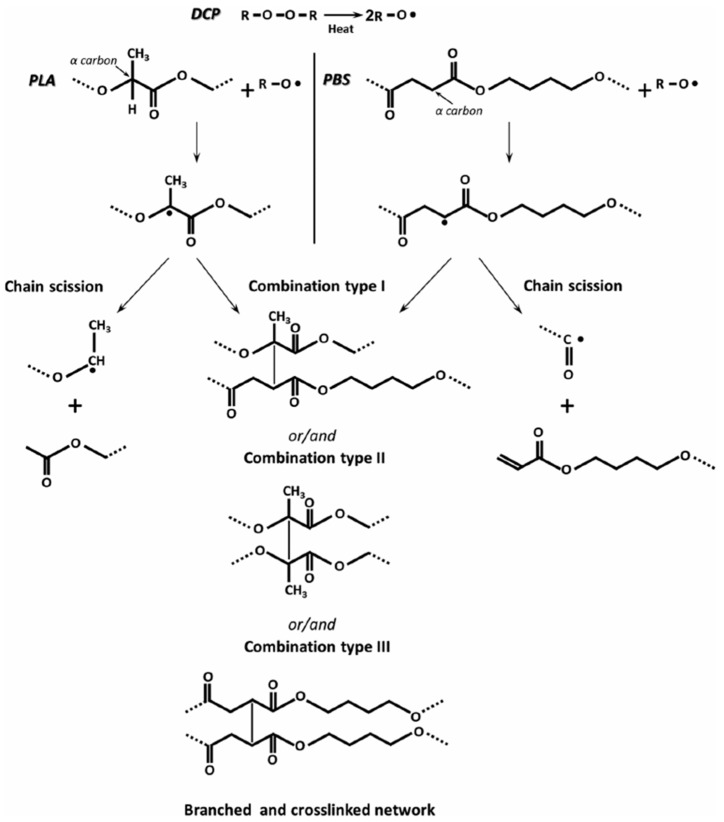
Mechanism of formation of a crosslinked network and chain scission between PLA and PBS by dicumyl peroxide (DCP) [44]. Reprinted from Polymer Testing, Volume 67, Srimalanon, P.; Prapagdee, B.; Markpin, T.; Sombatsompop, N., Effects of DCP as a free radical producer and HPQM as a biocide on the mechanical properties and antibacterial performance of in situ compatibilized PBS/PLA blends, 331–341, Copyright © 2018, with permission from Elsevier.

DCP has been introduced into PLA/PBS blends leading to cross-linked or branched structures by radical coupling reactions [16,44,48] (Figure 4). DCP contributes to the reduction of PBS domain sizes in the PLA matrix [16,44,48]. Wang et al. [16] reported that compression-molded sheets of a PLA/PBS (80/20) blend with DCP (0.1 phr) have the same elongation at break (250%) as the ones without DCP, while tensile modulus and strengths decrease, but the notched Izod impact strength increases about 8 times. Srimalanon et al. [44] reported that a PLA/PBS (80/20) blend achieves an elongation at break of more than 400% by loading 0.2 phr DCP; however, the tensile strength remains nearly independent of the DCP content. Ji et al. [48] observed the effects of DCP on the mechanical properties of PLA/PBS (80/20) blends. In presence of DCP (0.3 phr), the elongation at break rises from 49% to 195% and the tensile strength grows from 55 to 80 MPa [48]. At 0.2 and 0.3 phr DCP, Ji et al. [48] found the gel content of 3% and 6% respectively, in comparison with the high gel content (~40%) reported by Srimalanon et al. [44]. This may indicate the high sensitivity of peroxide compatibilized systems towards blend preparation conditions (temperature, mixing time, etc.). Moreover, DCP shortens the total isothermal crystallization time of the PLA/PBS (80/20) blend, indicating an increase in the crystallization rate [48].

Twice functionalized organoclay (TFC) with epoxy groups prepared by treating Cloisite 25A^®^ with (glycidoxypropyl)trimethoxy silane (GPTMS) has been used as a reactive compatibilizer for PLA/PBS (75/25) blends [49]. In the presence of 2 wt% TFC, the dispersed domain size is decreased from 1.8 to 0.59 µm [49]. The epoxy functional groups serve to link a chemical bond between TFC and the polymer components, which strengthens the interfacial interactions [49].

By using MCC, Joncryl^TM^ and preblended PLA/PBS systems, PLA/PBS/MCC (70/30/5/0.5) blends obtained improved toughness and higher thermal stability [24]. Joncryl^TM^ (0.5 phr) contributes to better miscibility between PLA and PBS resulting in higher impact strengths. That is because the epoxy groups of Joncryl^TM^ react with the carboxyl or hydroxyl groups of either PLA or PBS, leading to a long-chain branched structure of the polymers. The specimen preparation using the hot pressing of extruded granules might have allowed the additional time for the reaction of PLA/PBS/MCC blends with epoxy groups.

### 3.4. Copolymerization

The most representative non-reactive compatibilization method for polymer blends is the addition of block-copolymers [28]. The entanglement of a copolymer with blend components promotes strong interfacial adhesion which results in an improvement of the physical properties [50].

Supthanyakul et al. [23,37] reported the multi-functionality of random and block copolymers as a compatibilizer, plasticizer, and nucleating agent in PLA/PBS blends. Additionally, the film clarity of the blends is improved attributing to the contribution of the copolymers to the compatibility, crystallization rate, and spherulite formation [23,37]. Zhang et al. [45] observed the improvement of mechanical properties (e.g., elongation at break) by using linear block copolymers, three-arm-branched copolymers, and the combination of both types. The combination of linear copolymers PLLA-b-PBS-b-PLLA (20 phr) and branched copolymers (PLLA-b-PGMA)_3_ (2 phr) has shown a synergistic effect leading to an improvement of the elongation at break to 250% in PLLA/PBS (70/30) blends [45]. Using the linear and branched copolymers alone in the same amount only leads to an increase of elongation at break of 75% and 85%, respectively [45].

### 3.5. Discussion of Toughness Modification

The influencing factors for the toughness modification of PLA/PBS blends include the ratio of PLA and PBS, the type of the modifier, the ratio of polymer and modifier as well as the processing conditions. Despite the phase separation in the melt, PLA/PBS blends show some compatibility by simple blending. Among the different modification methods, reactive compatibilization is the most effective method for increasing the elongation at break. Less than 1 wt% benzoyl peroxide (BPO) and dicumyl peroxide (DCP) improve the PLA/PBS (80/20) blend toughness up to approximately 400%.The second most effective modification method is copolymerization, which represents a non-reactive compatibilization method. Combining linear block copolymer and branched copolymer has shown a synergetic effect on improving mechanical properties.

## 4. Crystallization Modification

Crystallizable PLA based blends need a balance between toughness and crystallization, especially in applications like blown films. The manufacturability [17], mechanical properties [51], clarity of films [12], and the biodegradability [52] depend on the characteristics of crystallization including the overall crystallinity, crystalline morphology, sizes of crystallites and their aggregates such as spherulites [51]. The effect of PBS, stereocomplexation, nucleation, and processing parameters on the crystallization of PLA/PBS blends are reviewed and described in detail in the following subchapters. Table 3 presents the main results from literature by order of the methods, ratios of PLA/PBS, and modifiers.

### 4.1. Effect of Poly(butylene succinate)

**Figure 5:**
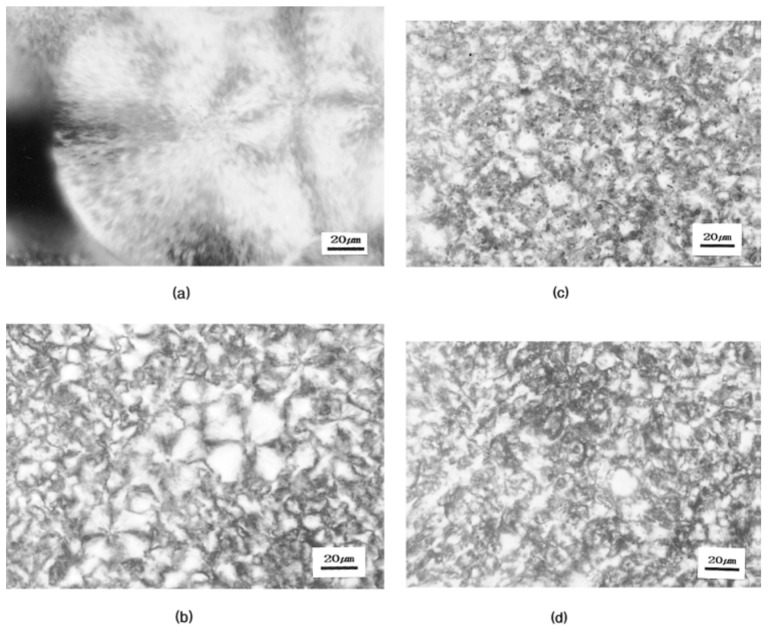
Polarized optical micrographs of PLA and PLA/PBS blends, isothermally crystallized at 100 °C for 12 h: (**a**) 100/0; (**b**) 90/10; (**c**) 80/20; (**d**) 70/30 [25]. Reprinted from Journal of Applied Polymer Science, Volume 86, Park, J.W.; Im, S.S., Phase behavior and morphology in blends of poly(L-lactic acid) and poly(butylene succinate), 647–655, Copyright © 2002, with permission from John Wiley and Sons.

**Figure 6:**
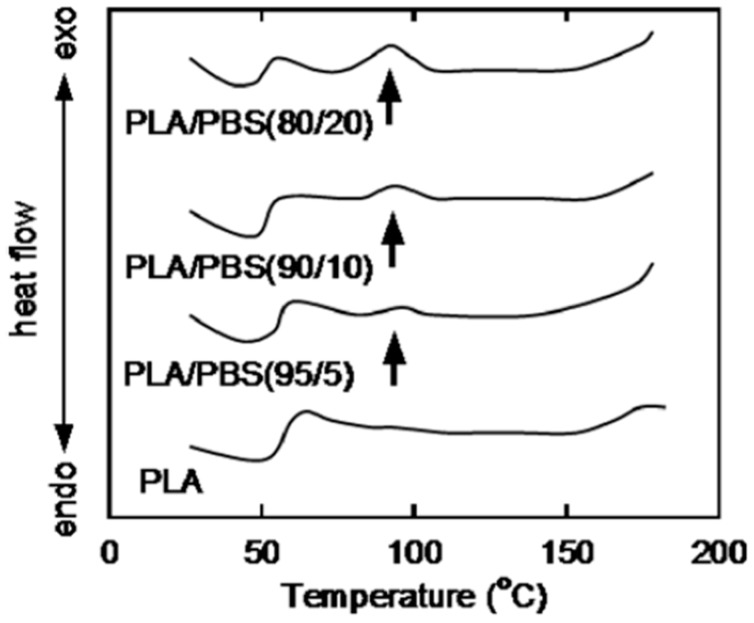
DSC cooling curves for PLA and PLA/PBS blends at cooling rate of 2 °C/min. The arrows in the figure indicate the crystallization peaks for PLA [22]. Reprinted from European Polymer Journal, Volume 44, Yokohara, T.; Yamaguchi, M., Structure and properties for biomass-based polyester blends of PLA and PBS, 677-685, Copyright © 2008, with permission from Elsevier.

Firstly, the influence of PBS on the crystallization of PLA based blends has been reviewed and it is observed that the crystallization rate of PLA is accelerated by PBS [16,22]. This is attributed to the nucleating effect of PBS at temperatures lower than its melting temperature [16,22]. This effect was detected by polarized optical microscopy (POM) at 100 °C within a cooling process (Figure 5) [25] and DSC at the cooling curve (Figure 6) [22]. As shown in the POM, the spherulites of pure PLA have a radially grown structure with an average radius of about 140 µm [25]. However, PLA/PBS blends containing less than 30 wt% PBS content demonstrate a large number of small spherulites that are much smaller and less regularly shaped [25]. This phenomenon was attributed to molten PBS droplets that act as crystallization nuclei for PLA [22]. Furthermore, PBS is said to induce an increase in the crystallinity of PLLA [38,53]. However, some other researchers indicate that PBS neither induces the crystallization [35] nor enhances the crystallinity of PLA [16,35]. The hindering effect of PBS on the total time for accomplishing the crystallization is observed in the isothermal crystallization at 110 °C [48]; this implies that the crystallization rate for PLA is hindered when it is blended with PBS alone [48].

### 4.2. Effect of Stereocomplexation

Stereocomplexation is a method for improving crystallization rate [55,56], heat resistance, and mechanical properties of PLA [56,57]. Stereocomplexation occurs upon blending PLLA and PDLA, which form a new crystalline structure through the interactions of stereoselective van der Waals forces [15].

D’Ambrosio et al. [14] reported the crystallization and stereocomplexation of PDLA and PLA-mb-PBS multiblock copolymers with molar mass below 10.000 g/mol. In the stereocomplex, an enhanced melting temperature (192.9 °C at 2nd heating) and a single glass transition temperature (−19.0 °C), due to the lower molar mass are observed. The PBS blocks with 30 wt% in this copolymer induce a higher crystallization rate of the stereocomplexes and three-dimensional spherulites. With increasing PBS content, the crystallinity and melting temperature of the stereocomplex decrease and the morphology of the stereocrystals changes to dendritic [14].

### 4.3. Effect of Nucleation

In order to increase the crystallization rate and crystallinity, nucleating agents can be added in the compounding process [17,54,58]. The crystallization temperature and the glass transition temperature are lowered, raising the nucleation density and improving the chain mobility [17]. Some additives acted successfully as nucleating agents in PLA/PBS blends, for instance: Talc [54], dicumyl peroxide (DCP), microcrystalline cellulose (MCC) [24], and LAK301 (an aromatic sulfonate derivate) [17].

The addition of talc to PLA/PBS (60/40) blends promotes the crystallization rate in isothermal crystallization at 95, 110, and 120 °C [54]. At 120 °C, PLA produces spherulites, while PBS should be in the molten state. The spherulite size decreases with increasing talc amount in blends [54].

The reactive compatibilizer DCP has been reported to affect the crystallization behavior of PLA/PBS (80/20) blends. Wang et al. [16] reported that PLA/PBS/DCP blends with ≥0.1 phr DCP prepared by a mixing chamber and then compression-molded into sheets became nearly amorphous; the PBS had no nucleating effect on the crystallization. However, Ji et al. [48] found that DCP shortens the total isothermal crystallization time, when PLA/PBS (80/20) are preblended using an internal mixer (1 min, at 180 °C) and subsequently DCP is added into the PLA/PBS blends for 9 min further blending, indicating an increase in the crystallization rate. The difference in both studies is probably due to the mixing method: Premixed PLA and PBS might have better interfaces for the crosslinking. The cross-linked and branched structure acts as nucleating sites which likely generate imperfect and small crystals for the reactive blends.

MCC has been used in combination with Joncryl^TM^ in PLA/PBS (70/30) blends [24]. MCC particles (5 phr) act as nuclei for PBS crystallization during cooling, while Joncryl^TM^ (0.5 phr) contributes to the better miscibility between PLA and PBS [24].

The commercial product LAK301, containing dimethyl 5-sulfoisophthalat/potassium salt, acts in PLA/PBS (67/33) blends as a nucleating agent and induces the crystallinity of PLA [17]. When the content of LAK301 increases, films achieve better mechanical isotropy in machine and cross directions while the tensile properties decrease, and the tear propagation resistance improves [17].

### 4.4. Effect of Processing Parameters

On the one hand, crystallization is important for processing. Good crystallization allows the rapid and efficient cutting of the extruded strand (thus easily producing pellets). Furthermore, in blown film extrusion, it influences the process stability and the film stiffness [17]. On the other hand, the processing parameters have also been reported to influence the crystallization of PLA based blends [39,59]. In blown film production, it is of high importance to investigate the processing parameters such as the blow-up ratio (BUR), the draw down ratio (DDR), and the forming ratio (FR) [17]. The biaxial stretching in the film blowing process governs the orientation of polymer chains which in turn induces increased crystallinity leading to enhanced barrier properties and chemical inertness [60]. Liu et al. [39] reported an increase in the degree of crystallinity of about 5% by the biaxial stretching of a PLA/PBS (90/10) film (draw ratio 4 × 4) using a film stretcher machine (at 85 °C) [39]. Additionally, the modulus of elasticity was improved for approximately 1 GPa applying a draw ratio of 3 × 3 [39].

### 4.5. Discussion of the Crystallization Modification

The blend partner PBS, stereocomplexation, nucleation, as well as processing parameters affect the crystallization behavior of PLA-based blends such as crystallinity, crystalline morphology, sizes of crystallites and their aggregates such as spherulites. PBS has shown both promoting and hindering effects on the crystallization rate of PLA in different research. The stereocomplexation of PDLA and PLLA_70_-mb-PBS_30_ multiblock copolymers results in a higher crystallization rate and an increase of melting temperature. The reactive compatibilizer dicumyl peroxide (DCP) (0.3 phr) was found to increase the crystallization rate of PLA in isothermal crystallization at 110 °C. Furthermore, biaxial stretching in the film production can induce increased crystallinity (for 4%) and modulus of elasticity due to the orientation of polymer chains.

## 5. Degradation

While achieving improved toughness and crystallization, the modified PLA/PBS blends should reserve the biodegradability [43].

### 5.1. Standards for Biodegradation and Disintegration of Plastics

According to the Standard ISO 16929-2013, biodegradation is caused by biological activity especially by enzymatic action leading to a significant change in the chemical structure of a material, while disintegration is the physical breakdown of material into very small fragments. For compostable materials, it is required to achieve a high degree of biodegradation and disintegration on specified limited time-scales under composting conditions, without any harmful effect on the composting process or compost quality [28].

A distinction needs to be made between industrial and home composting [61]. Industrial composting conditions are characterized by elevated temperatures (55–60 °C) combined with high relative humidity, the presence of oxygen, and periodical mixing. It takes place under given and predictable conditions. Home-composting, on the other hand, usually means uncontrolled conditions, depending on a great extent on the geographical and climatological situation as well as on individual actions taken by households [61]. Neat PLA needs industrial composting conditions for quick biodegradation [19,62], and will not biodegrade within a reasonable time frame in soil or home compost, unless special measures (e.g., blending, copolymerization) are applied [19]. Under controlled condition (58 °C, 65% RH), PLLA biodegrades faster than PBS. However, PBS has the ability to biodegrade at lower temperatures (<35 °C) [19,62].

Worldwide, standards and draft standards for biodegradability and disintegration of plastics have been developed (Table 4). However, there is no general standard for biodegradability of plastics in an open and uncontrolled environment, since biodegradability depends on diverse parameters.

### 5.2. Influencing Factors and Degradation Mechanisms

On the one hand, the biodegradability of polymers depends strongly on the environmental conditions; more important are: Temperature, the presence of microorganisms, oxygen, and water [61]. On the other hand, biodegradability is in first place influenced by the chemical structure of the polymers. Microorganisms or their enzymes, in general, cannot easily attack polymers with a C–C backbone (i.e., polyolefins).

Additionally, for polymers with principally degradable backbone chains, e.g., polyesters, the material structure on a nano- and micro-scale, like crystallinity, orientation, and morphology of blends, influences biodegradation. Among these, the degree of crystallinity is the major factor [52]. Additionally, the shape of the plastics part (e.g., thickness of a film) affects the disintegration results of slowly degrading plastics such as PLLA and PBS [63].

Regarding the biodegradation mechanisms, there are differences between enzymatic chain scissions and non-enzymatic mechanisms, especially in hydrolytic degradation (Table 5). Because the enzymes cannot reach inside the specimen, enzymatic degradation proceeds only on the polymers’ surface. Amorphous or less-ordered regions degrade more easily than crystalline regions. During the enzymatic degradation, there is no significant measurable change in the molecular weight of a specimen. Only polymers on the surface degrade and the degradation products with low molecular weights are removed from the system by dissolution in the surrounding aqueous medium or by the uptake of microorganisms adhered to the surface [64].

In slow bulk degradation processes caused by chemical reactions like hydrolysis, small catalysts (e.g., organic acids) and reagents (here, water) diffuse into polymer systems. The crystallinity, cross-linking, and other morphological properties of the polymers influence the rate of this process. In the first stage, there is no weight loss. Later, the average molecular weight throughout the plastic part decreases by random chain splitting which causes a reduction in mechanical properties such as tensile strength, elongation at break, and impact strength. Following in the final stage, morphological breakdown occurs with the fragmentation of plastics parts [64].

**Figure 7:**
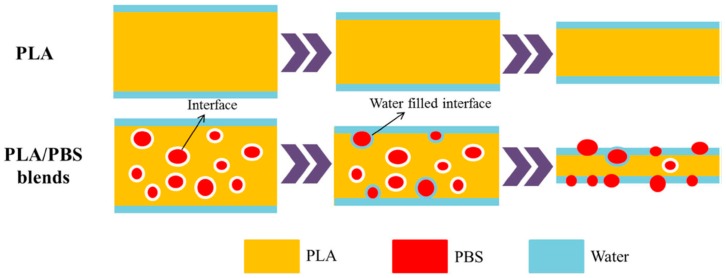
Schematic representations for the hydrolytic degradation process of pure PLA and PLA/PBS blends [35]. Reprinted by permission from Springer Nature: Polymer Bulletin, Accelerated hydrolytic degradation of poly(lactic acid) achieved by adding poly(butylene succinate), Wang, Y.-P.; Xiao, Y.-J.; Duan, J.; Yang, J.-H.; Wang, Y.; Zhang, C.-L., Copyright © Springer-Verlag Berlin Heidelberg 2015.

Figure 7 shows the hydrolytic degradation behavior of pure PLA and PLA/PBS blends. Pure PLA degradation proceeds mainly via a surface-erosion mechanism, and the sample size and shape gradually change from the initial stage to the later stage [35]. In spite of the wettability enhancement by the presence of PBS component, PLA/PBS blends initially exhibit a similar hydrolytic degradation via surface-erosion like pure PLA. Once PBS particles are increasingly exposed, water has the chance to penetrate into the gaps between the PLA matrix and PBS particles. Consequently, the hydrolytic degradation process of the PLA matrix, too, is accelerated [35].

### 5.3. Biodegradation and Disintegradation of PLA/PBS Blends

The blend composition and the possible presence of compatibilizers or fillers can interact with each other during degradation [65]. This can strongly influence the degradation behavior of PLA/PBS blends so that the modified or unmodified PLA/PBS blends differ from the neat components in respect of their degradation behavior. Table 6 gives an overview of the studies of biodegradation and disintegration of PLA/PBS blends are summarized by the degradation type.

Hydrolytic degradation tests of PLA/PBS blends were carried out in NaOH solution [35], which mainly leads to changes on the surface of samples. The interior begins to degrade with increasing time. The addition of the immiscible PBS component into PLA provides permeable channels in the blend material for water penetration. This enhances the wettability of the sample surface so that PLA/PBS blends exhibit higher hydrophilicity than neat PLA [35].

In another research group, soil burial tests were performed to investigate the effect of the ambient environment on the blends. The weight loss of blends after soil burial demonstrates that PLA/PBS blends with higher content of PBS have a higher rate of disintegration within 60 days (weight loss: approx. 6% in the PLA/PBS (80/20) blend, approx. 12% in the PLA/PBS (20/80) blend) [66]. Gel permeation chromatography was used to determine the molecular weight distribution of the polymer chains in the blend samples. The number average molecular weight decreases as a function of degradation time due to the chemical hydrolysis of PLA and PBS [66].

**Figure 8:**
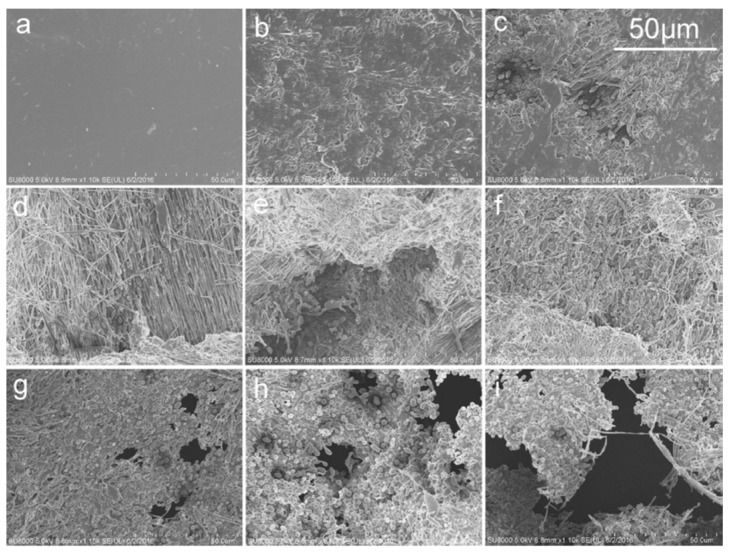
SEM surface structure of PBS/PLA/benzoyl peroxide (BPO) films degraded by proteinase K. (**a**) 0 h; (**b**) 12 h; (**c**) 24 h; (**d**) 36 h; (**e**) 48 h; (**f**) 60 h; (**g**) 72 h; (**h**) 84 h; (**i**) 96 h [43]. Reprinted by permission from Springer Nature: Polymer Bulletin, Blending modification of PBS/PLA and its enzymatic degradation, Hu, X.; Su, T.; Li, P.; Wang, Z., Copyright © Springer-Verlag Berlin Heidelberg 2017.

The enzymatic degradation of PLA/PBS blend films modified by benzoyl peroxide (BPO) was researched with proteinase K for 96 h (Figure 8) [43]. The films present a smooth surface before degradation. During the degradation, filaments occur more and more clearly and even emerge as segments. In the course of the degradation, films become increasingly thin until some holes appear. Randomly distributed holes form into large ones. The degradation rate of the blend reaches 67% after 96 h of incubation [43].

The disintegration of an 80/20 PLA/PBS blend film and its nanocomposite with modified and unmodified cellulose nanocrystals, both prepared by solution casting, have been studied in aerobic industrial composting conditions (58 °C, 50% RH) [53]. PBS reduces the degradation rate as a consequence of higher PLA crystallinity induced by PBS. The presence of surfactant-modified cellulose nanocrystals facilitates the disintegration of the blends. Nevertheless, a degree of disintegration exceeding 90% after 17 days is reached by all samples [53].

## 6. Recycling

The plastics recycling industry is increasingly aware of the growing market for bioplastics products. Due to the biodegradability, the products of PLA/PBS blends are able to biodegrade and convert into carbon dioxide and water at certain conditions. From the author’s point of view, this could be a favorable possibility for some plastic parts, e.g., mulch films. However, biodegradation or composting neither contributes to material recycling nor to energy recovery.

Chemical recycling is an effective method for PLA/PBS blends. During this process, polymer chain molecules are broken down into small molecules (e.g., monomers), which can be re-fed to polymerization reactions [67]. Tsuneizumi et al. [68] have conducted chemical recycling of PLA/PBS blends using two routes. The first route makes use of the separation of PLA and PBS by their solubility in toluene. The other route is based on the sequential degradation of PLA/PBS blends using a lipase first to degrade PBS into cyclic oligomer. The oligomers were repolymerized to produce new PBS. Next, PLLA was degraded into repolymerizable lactide oligomers [68].

## 7. Application

Bio-based and biodegradable PLA and PBS with low toxicity have potential in food packaging, biomedicine, and agricultural markets [69]. Commercial PLA/PBS blends have been utilized for food service ware production by NatureWorks LLC [70]. Based on extensive research, PLA/PBS blends have been successfully modified and processed into fibers, blown films, flat films, and sheets. The blends (e.g., as stretchable films plasticized with isosorbide [41]) have been suggested as a novel solution for food packaging applications [41,53]. Moreover, FKuR Kunststoff GmbH and Fraunhofer UMSICHT developed the highly bio-based and compostable product Bio-Flex^®^ S 5630 made of PLA/PBS blends, which can be used for flat sheet extrusion with subsequent thermoforming as well as injection molding [71,72].

## 8. Conclusions

This brief review summarizes literature information on the toughness and crystallization behavior, degradation, recycling, and the applications of PLA/PBS blends.

To modify the PLA toughness, PBS is applied. Methods include simple blending, plasticization, reactive compatibilization and copolymerization. PLA and PBS are immiscible based on the experimental results, therefore blend properties depend strongly on preparation conditions. SEM micrographs demonstrate the biphasic morphology of their blends. A study of the influence of molecular weight on miscibility will be worthy for the correlation of morphological and mechanical properties. PLA is predominantly used as a polymer matrix while PBS acts as a dispersed component. Simple blending PLA with PBS will increase the elongation at break but decrease tensile strength and modulus compared to neat PLA. Enhanced ductility of PLA-based blends will be reached via plasticization due to reduced intermolecular forces and improved mobility of the polymer chains. Only a few studies were reported about the plasticization. The results demonstrate the expected effect of plasticizers to tensile modulus, strength, and elongation at break. A lot of studies were done in reactive compatibilization improving the toughness in a more balanced way. Among the different reactive compatibilizers, DCP shows the highest effectiveness. However, it is important to pay attention to the gelation which can restrict the applicability. In comparison with reactive compatibilization, a moderate improvement on elongation at break is reachable by copolymerization as a non-reactive compatibilization method. Additionally, copolymers have shown the plasticizing and nucleating effect for PLA/PBS blends. A synergetic effect on the properties improvement is achieved by combining linear block and branched copolymers.

To modify the PLA crystallization in PLA/PBS blends, the effect of PBS, stereocomplexation, nucleation, and processing parameters on the crystallization behavior are reviewed. PBS exhibits both promoting and hindering effects on the crystallization rate of PLA. A high crystallization rate can be achieved by using DCP in isothermal crystallization. The stereocomplexation of PDLA and PLLA_70_-mb-PBS_30_ multiblock copolymers causes a higher crystallization rate, a higher melting temperature, and a single glass transition temperature. The right nucleating agent can markable improve crystallinity, crystallization rate and size of spherulites. Furthermore, biaxial stretching induces increased crystallinity and tensile modulus in the film products due to the polymer chain orientation.

The degradation of PLA/PBS blends is reviewed regarding the standards, influencing factors, and degradation mechanisms. PLA needs industrial composting conditions for quick biodegradation, and cannot biodegrade within a reasonable time frame in soil or home compost without any special measure. However, PBS biodegrades at less than 35 °C. Immiscible PBS particles induce gaps in blends, providing channels for water penetration. During the degradation, the weight loss increases and the number-average molecular weight decreases due to chemical hydrolysis of PLA and PBS.

Chemical recycling is a promising end-of-life option for PLA/PBS blends. Polymers are broken down into small molecules that are re-fed to polymerization reactions. One route uses the separation of PLA and PBS by their solubility in toluene. Another route based on the sequential degradation of PLA/PBS blends using a lipase. Firstly, PBS is degraded into cyclic oligomers, that are repolymerized to produce new PBS. Then PLA is degraded into lactide oligomers. The biodegradation of PLA/PBS blends could be a favorable option for applications like mulch film. However, biodegradation neither contributes to material recycling nor to energy recovery.

PLA/PBS blends have been successfully processed into fibers, sheets, blown and flat films. Products made of them are commercially available for food service goods. Modified PLA/PBS blends with improved properties might have a great future ahead of them in the biomedicine and agricultural markets.

## Figures and Tables

**Table 1:** Material properties of polylactide (PLA) and PBS, adapted from [19,20,21].

Property	PLA	PBS
Glass transition temperature [°C]	55–60	−32
Melting point * [°C]	150–175	114
Heat distortion temperature [°C]	55	97
Modulus of elasticity [MPa]	3500–4150	550–700
Tensile strength [MPa]	50–70	34
Elongation at break [%]	4–7	560
Derived from renewable raw materials	Yes	Partly
Biodegradable at 70 °C	Yes	Yes
Biodegradable at 30 °C	No	Yes

Legend: *: the melting point of PLA depends strongly on the composition of stereoisomers.

**Table 2:** Toughness modification.

Method	Ratio PLA/PBS	Modifier	Ratio Polymer/Modifier	Test Specimen	Morphology, Crystallization, and Mechanical Properties	Ref
Plasticiz.	80/20	Isosorbide diester (ISE)	100:15	Extruded flat films, 180–205 °C, 787 rpm, thickness: 20–50 µm	Porous structure ↓; presence of holes and defects ↓; cold crystalliz. temp.↓ for 21 °C; *Tg* ↓ from 55.3 to 34.7 °C; elongation at break: 250%. *MOE*: 511 MPa; *TS*: 19.8 MPa.	[41]
Plasticiz.	80/20	Poly (ethylene glycol) (PEG)	100:10	Injection-molded dumbbell at 220 °C, thickness: 3 mm	Tg of PLA ↓ significantly, crystallization rate of PLA ↓, elongation at break ↑ slightly.	[36]
Reactive compat.	90/10	Lysine triisocyanate (LTI)	100:0.5	Injection-molded samples at 220 °C, mold: 30 °C, 40–60 MPa, *^1^	Particle number ↑; particle size ↓; elongation at break ↑ to >150%. impact strength ↑ 3-fold than that of PLA.	[42]
Reactive compat.	80/20	Benzoyl peroxide (BPO)	100:1	Hot-pressed sheets, at 160 °C, 3 min, then cold-pressed at room temperature, thickness: 0.5 mm	No phase separation; smooth surface; crystallinity ↓ due to cross-linking; elongation at break ↑ to about 400%, *TS*: little change.	[43]
Reactive compat.	80/20	Dicumyl peroxide (DCP)	100:0.3	Pressed tensile bars, at 180 °C, 10 MPa, thickness: 0.6 mm	PBS domain size ↓; isothermal crystallization time ↓; *TS* ↑ from 55 to 80 MPa, elongation at break ↑ from 49 to 205%.	[48]
Reactive compat.	80/20	Dicumyl peroxide (DCP)	100:0.2	Injection-molded specimens, cylinder 170–200 °C, die: 195 °C, mold: 25 °C, *^1^	PBS domain size ↓; improved dispersion of PBS in PLA matrix; *TS* & *MOE* slightly ↑; elongation at break ↑ >400%.	[44]
Reactive compat.	80/20	Dicumyl peroxide (DCP)	100:0.1	Compression-molded sheets, thickness: 1 mm, at 170 °C, 8 min	PBS domain size ↓; uniform morphology; no nucleating effect on PLLA; elongation at break (250%): no change; impact strength ↑ for 8 times.	[16]
Reactive compat.	70/30	Methylene diphenyl diisocyanate (MDI)	100:5	Compression-molded tensile bars, at 180 °C, mold: 50 °C, 5 MPa, *^1^	Morphology: uniform, no cavities or obvious phase separation *TS* & *MOE* ↓ slightly; elongation at break ↑ to 285%.	[46]
Reactive compat.	75/25	Twice functionalized organoclay (TFC) with epoxy groups	100:2	Hot pressed specimens: 10 mm × 50 mm × 1 mm	PBS domain size ↓ from 1.8 to 0.59 µm, when TFC at 2 phr; tensile modulus ↑ from 1075 to 1407 MPa; elongation at break ↑ from 72 to 76%.	[49]
Reactive compat.	50/50	Glycidyl methacrylate (GMA)	100:10	Compression-molded sheets, 0.25 mm thick, 120 bar, 140–190 °C	Fibril formation before tensile fracture of the sheets; miscibility ↑; crystallinity ↓ due to crosslinking; elongation at break ↑ >4000 times.	[47]
Copolym.	80/20	Random copolymer rPBSL	100:5	Blown film, thickness: 40 µm, extruder: 100–175 °C, 80–100 rpm	Smooth surface without droplets; *Tg* ↓ from 52 to 43 °C; *Tc* ↓ from 103 to 85 °C; crystallinity ↑ from 22 to 34%; spherulite growth rate ↑; elongation at break ↑ for 4 times.	[23]
Copolym.	80/20	Linear block copolymer PLLA_40_-b-PBS_28_-b-PLLA_40_	100:3	Compression-molded film, thickness: 40 µm	Smooth surface; formation of uniform spherulites; isothermal crystallization ↑; Transmittance (clarity) ↑; *Tg* ↓ to 20 °C; elongation at break ↑ for 2-fold.	[37]
Copolym.	70/30	Linear block copolymer PLLA_37.5_-b-PBS_25_-b-PLLA_37.5_	100:20	Compression-molded sheets, at 180 °C, 10 MPa, thickness: 1 mm	PBS domain size ↓; elongation at break ↑ from 26% to 75%.	[45]
Copolym.	70/30	3-arm block copolymer (PLLA-b- PGMA)_3_	100:2	Compression-molded sheets at 180 °C, 10 MPa, thickness: 1 mm	PBS domain size ↓; uniform surface; cavities almost disappeared; elongation at break ↑ from 26% to 85%.	[45]
Copolym.	70/30	Linear block copolymer + branched copolymer	100:20:2	Compression-molded sheets, at 180 °C, 10 MPa, thickness: 1 mm	No obvious phase separation, uneven fracture surface; elongation at break ↑ up to 250%.	[45]

Legend: *MOE*: modulus of elasticity; *TS*: tensile strength; *Tc*: crystallization temperature; *Tg*: glass transition temperature; *^1^: no specification thickness given.

**Table 3:** Crystallization modification.

Method	Ratio PLA/PBS	Modifier	Ratio Polymer/Modifier	Test Specimen	Morphology, Crystallization, and Mechanical Properties	Ref
Stereo-com.	100/0	PLLA_70_-mb-PBS_30_ multiblock copolymer for PDLA-matrix	47:53	Solution-blending and drying; casting on petri-dishes	Negative 3-dimensional spherulites in SEM; a single *Tg* (−19 °C): miscibility ↑; enhanced *Tm* = 192.9 °C at 2nd heating; crystallization rate ↑.	[14]
Nucleat.	60/40	Talc with an average particle size of 1 µm	100:5	Casting on petri-dishes; solvent (chloroform) was removed in oven at 80 °C, 24 h	120 °C: spherulite size ↓ as talc [%] ↑. 90 °C: spherulite size ↑ as talc [%] ↑. Crystallization rate ↑ at 95 °C, 110 °C, and 120 °C in isothermal crystallization.	[54]
Reactive compat. + Nucleat.	80/20	Dicumyl peroxide (DCP)	100:0.3	Hot and then cold pressed tensile bars, at 180 °C, 10 MPa, thickness: 0.6 mm	PBS domain size ↓; total isothermal crystallization time ↓; *TS* ↑ from 55 to 80 MPa; elongation at break ↑ from 49 to 205%.	[48]
Reactive compat. + Nucleat.	70/30	Microcrystalline cellulose; epoxides (Joncryl^TM^)	100:5:0.5	Hot pressed sheets: thickness: 1 mm and 3 mm, at 180 °C, 7 min	Better miscibility in SEM; no *T_cc_* of PBS in blend; MCC promotes PBS crystallization; impact strength ↑ from 6 to 13 KJ/m^2^; elongation at break ↓.	[24]
Reactive compat. + Nucleat.	67/33	EJ400 + LAK 301 (aromatic sulfonate derivate)	(90:10):3	Blown film, blow-up ratio: 4.75; 140–190 °C, 200 rpm; thickness: 40 µm	Nucleating agent agglomerates; crystallinity of PLA ↑ by LAK; when LAK content ↑: more isotropy of films (MD/CD) in tensile test; tear resistance ↑.	[17]

Legend: *Tcc*: cold crystallization temperature; *Tg*: glass transition temperature; *Tm*: melting temperature; TS: tensile strength.

**Table 4:** Extract of latest standards and drafts in respect of biodegradability and disintegration.

(Draft) Standard	Title
prEN ISO 14851:2016	Determination of the ultimate aerobic biodegradability of plastic materials in an aqueous medium—method by measuring the oxygen demand in a closed respirometer
prEN ISO 14852:2017	Determination of the ultimate aerobic biodegradability of plastic materials in an aqueous medium—method by analysis of evolved carbon dioxide
EN ISO 14855-1:2012	Determination of the ultimate aerobic biodegradability of plastic materials under controlled composting conditions—method by analysis of evolved carbon dioxide—Part 1: General method
prEN ISO 14855-2:2017	Determination of the ultimate aerobic biodegradability of plastic materials under controlled composting conditions—method by analysis of evolved carbon dioxide—Part 2: Gravimetric measurement of carbon dioxide evolved in a laboratory-scale test
EN 17033:2018	Plastics—Biodegradable mulch films for use in agriculture and horticulture—Requirements and test methods
prEN ISO 17556:2018	Plastics—Determination of the ultimate aerobic biodegradability of plastic materials in soil by measuring the oxygen demand in a respirometer or the amount of carbon dioxide evolved
EN ISO 20200:2015	Plastics—Determination of the degree of disintegration of plastic materials under simulated composting conditions in a laboratory-scale test
ISO 16929:2013-04	Plastics—Determination of the degree of disintegration of plastic materials under defined composting conditions in a pilot-scale test

**Table 5:** The first stage of biodegradation of PLA and PBS, adapted from [64]. Reprinted from Polymers for Advanced Technologies, Volume 8, Mochizuki, M.; Hirami, M., Structural Effects on the Biodegradation of Aliphatic Polyesters, 203-209, Copyright © 1997, with permission from John Wiley and Sons.

Polymer	PLA	PBS
Type	Chemical hydrolysis	Enzymatic hydrolysis
Access point	Outer to inner	Outer only
Surface appearance	Smooth (not eroded)	Rough (eroded)
Weight loss	Negligible	Detectable
Molecular weight reduction	Detectable	Negligible

**Table 6:** Biodegradation and disintegration of PLA/PBS blends.

Type	Ratio PLA/PBS	Test Specimen	Degradation Conditions	Degradation Outcomes	Ref
Disintegration in composting conditions	80/20	Films prepared by solvent casting: 15 × 15 × 0.03 mm^3^	4–6 cm depth in boxes with soil: aerobic, 58 °C, 50% RH, 17 days	The disintegration value is reduced as a consequence of higher crystalline nature induced by PBS; surfactant facilitates the disintegration. degree of disintegration >90%, 17 days.	[53]
Enzymatic degradation	80/20/1 (BPO)	Films 30 × 10 × 0.1 mm^3^	Incubation: 37 °C, in buffer (pH = 8), proteinase K	Filaments appear on the surface; films became thin; randomly distributed holes form into large ones; degradation rate: 67%, 96 h.	[43]
Hydrolytic degradation (Soil burial test)	80/20 etc.	Compression-molded sheets: at 180 °C, 12 MPa, 0.3 mm thick.	Soil temp.: 29–39 °C; soil moisture: 18–30%, 60 days	Blends with higher content of PBS have higher rate of biodegradation; *M_n_* decreases as a function of degradation time.	[66]
Hydrolytic degradation (NaOH solution)	70/30	Compression-molded sheets (at 190 °C, 5 MPa, 50 µm thick)	Incubation: 37 °C, NaOH, pH = 13	Immiscible PBS particles induce gaps in blends, providing channels for water penetration; hydrolytic degradation ↑; weight loss per unit area ↑ when PBS content ↑.	[35]

Legend: BPO: benzoyl peroxide; RH: relative humidity; *M_n_*: number-average molecular weight.

## References

[1] United Nations About the Sustainable Development Goals. //www.un.org/sustainabledevelopment/sustainable-development-goals/.

[2] The RTO Innovation Summit Impact through Research: Applied Research for Europe’s Future. https://www.fraunhofer.de/en/press/research-news/2018/november/the-rto-summit.html.

[3] European Bioplastics (2018). Plastics Strategy—Contribution of Bioplastics to a Sustainable Circular Plastics Economy.

[4] European Bioplastics Bioplastics Market Data. https://www.european-bioplastics.org/market/.

[5] Wang L., Ma W., Gross R.A., McCarthy S.P. (1998). Reactive compatibilization of biodegradable blends of poly(lactic acid) and poly(ε-caprolactone). Polym. Degrad. Stab..

[6] Sheth M., Kumar R.A., Davé V., Gross R.A., Mccarthy S.P. (1997). Biodegradable polymer blends of poly(lactic acid) and poly(ethylene glycol). J. Appl. Polym. Sci..

[7] Zhao P., Liu W., Wu Q., Ren J. (2010). Preparation, Mechanical, and Thermal Properties of Biodegradable Polyesters/Poly(Lactic Acid) Blends. J. Nanomater..

[8] Jiang L., Wolcott M., Zhang J. (2006). Study of biodegradable polylactide/poly/butylene adipate-co-terephthalate) blends. Biomacromolecules.

[9] Bhatia A., Gupta R., Bhattacharya S., Choi H. (2007). Compatibility of biodegradable poly (lactic acid) (PLA) and poly (butylene succinate) (PBS) blends for packaging application. Korea-Aust. Rhelogy J..

[10] Gigli M., Fabbri M., Lotti N., Gamberini R., Rimini B., Munari A. (2016). Poly(butylene succinate)-based polyesters for biomedical applications: A review. Eur. Polym. J..

[11] Changwichan K., Silalertruksa T., Gheewala S. (2018). Eco-Efficiency Assessment of Bioplastics Production Systems and End-of-Life Options. Sustainability.

[12] Hamad K., Kaseem M., Ayyoob M., Joo J., Deri F. (2018). Polylactic acid blends: The future of green, light and tough. Prog. Polym. Sci..

[13] Novamont Mater-Biotech: Inaugurated the World First Plant for the Production of Bio-Butanediol from Renewable Resources. https://www.novamont.com/eng/read-news/mater-biotech/.

[14] D’Ambrosio R., Michell R., Mincheva R., Hernández R., Mijangos C., Dubois P., Müller A. (2018). Crystallization and Stereocomplexation of PLA-mb-PBS Multi-Block Copolymers. Polymers.

[15] Ikada Y., Jamshidi K., Tsuji H., Hyon S.H. (1987). Stereocomplex formation between enantiomeric poly(lactides). Macromolecules.

[16] Wang R., Wang S., Zhang Y., Wan C., Ma P. (2009). Toughening modification of PLLA/PBS blends via in situ compatibilization. Polym. Eng. Sci..

[17] Mallegni N., Phuong T.V., Coltelli M.-B., Cinelli P., Lazzeri A. (2018). Poly(lactic acid) (PLA) Based Tear Resistant and Biodegradable Flexible Films by Blown Film Extrusion. Materials.

[18] Zhang K., Mohanty A.K., Misra M. (2012). Fully biodegradable and biorenewable ternary blends from polylactide, poly(3-hydroxybutyrate-co-hydroxyvalerate) and poly(butylene succinate) with balanced properties. ACS Appl. Mater. Interfaces.

[19] Weise B., Huysman S., Manvi P., Theunissen L. (2018). PBS-based Fibres for Renewable Textiles. Bioplatics Mag..

[20] Xu J., Guo B.-H. (2010). Poly(butylene succinate) and its copolymers: Research, development and industrialization. Biotechnol. J..

[21] Farah S., Anderson D.G., Langer R. (2016). Physical and mechanical properties of PLA, and their functions in widespread applications—A comprehensive review. Adv. Drug Deliv. Rev..

[22] Yokohara T., Yamaguchi M. (2008). Structure and properties for biomass-based polyester blends of PLA and PBS. Eur. Polym. J..

[23] Supthanyakul R., Kaabbuathong N., Chirachanchai S. (2016). Random poly(butylene succinate-co-lactic acid) as a multi-functional additive for miscibility, toughness, and clarity of PLA/PBS blends. Polymer.

[24] Chaiwutthinan P., Pimpan V., Chuayjuljit S., Leejarkpai T. (2015). Biodegradable Plastics Prepared from Poly(lactic acid), Poly(butylene succinate) and Microcrystalline Cellulose Extracted from Waste-Cotton Fabric with a Chain Extender. J. Polym. Environ..

[25] Park J.W., Im S.S. (2002). Phase behavior and morphology in blends of poly(l-lactic acid) and poly(butylene succinate). J. Appl. Polym. Sci..

[26] Park S.B., Hwang S.Y., Moon C.W., Im S.S., Yoo E.S. (2010). Plasticizer effect of novel PBS ionomer in PLA/PBS ionomer blends. Macromol. Res..

[27] Kfoury G., Raquez J.-M., Hassouna F., Odent J., Toniazzo V., Ruch D., Dubois P. (2013). Recent advances in high performance poly(lactide): From “green” plasticization to super-tough materials via (reactive) compounding. Front. Chem..

[28] Imre B., Pukánszky B. (2013). Compatibilization in bio-based and biodegradable polymer blends. Eur. Polym. J..

[29] Hansen C.M. (2007). Hansen Solubility Parameters. A User’s Handbook.

[30] Abbott S., Auras R., Lim L.-T., Selke S.E.M., Tsuji H. (2010). Chemical compatibility of poly(lactic acid): A practical framework using hansen solubility parameters. Poly(Lactic Acid): Synthesis, Structures, Properties, Processing, and Applications.

[31] Ruellan A., Guinault A., Sollogoub C., Ducruet V., Domenek S. (2015). Solubility factors as screening tools of biodegradable toughening agents of polylactide. J. Appl. Polym. Sci..

[32] Van Krevelen D.W., Nijenhuis K.T. (2009). Properties of Polymers. Their Correlation with Chemical Structure Their Numerical Estimation and Prediction from Additive Group Contributions, 4th completely rev. ed..

[33] Valerio O., Misra M., Mohanty A.K. (2018). Statistical design of sustainable thermoplastic blends of poly(glycerol succinate-co-maleate) (PGSMA), poly(lactic acid) (PLA) and poly(butylene succinate) (PBS). Polym. Test..

[34] Robeson L.M. (2007). Polymer Blends. A Comprehensive Review.

[35] Wang Y.-P., Xiao Y.-J., Duan J., Yang J.-H., Wang Y., Zhang C.-L. (2016). Accelerated hydrolytic degradation of poly(lactic acid) achieved by adding poly(butylene succinate). Polym. Bull..

[36] Pivsa-Art W., Fujii K., Nomura K., Aso Y., Ohara H., Yamane H. (2016). The effect of poly(ethylene glycol) as plasticizer in blends of poly(lactic acid) and poly(butylene succinate). J. Appl. Polym. Sci..

[37] Supthanyakul R., Kaabbuathong N., Chirachanchai S. (2017). Poly(l-lactide-b-butylene succinate-b-l-lactide) triblock copolymer: A multi-functional additive for PLA/PBS blend with a key performance on film clarity. Polym. Degrad. Stab..

[38] Zhang X., Liu Q., Shi J., Ye H., Zhou Q. (2018). Distinctive Tensile Properties of the Blends of Poly(l-lactic acid) (PLLA) and Poly(butylene succinate) (PBS). J. Polym. Environ..

[39] Liu X., Dever M., Fair N., Benson R.S. (1997). Thermal and Mechanical Properties of Poly(lactic Acid) and Poly(ethylene/butylene Succinate) blends. J. Polym. Environ..

[40] Vieira M.G.A., da Silva M.A., dos Santos L.O., Beppu M.M. (2011). Natural-based plasticizers and biopolymer films: A review. Eur. Polym. J..

[41] Fortunati E., Puglia D., Iannoni A., Terenzi A., Kenny J.M., Torre L. (2017). Processing Conditions, Thermal and Mechanical Responses of Stretchable Poly (Lactic Acid)/Poly (Butylene Succinate) Films. Materials.

[42] Harada M., Ohya T., Iida K., Hayashi H., Hirano K., Fukuda H. (2007). Increased impact strength of biodegradable poly(lactic acid)/poly(butylene succinate) blend composites by using isocyanate as a reactive processing agent. J. Appl. Polym. Sci..

[43] Hu X., Su T., Li P., Wang Z. (2018). Blending modification of PBS/PLA and its enzymatic degradation. Polym. Bull..

[44] Srimalanon P., Prapagdee B., Markpin T., Sombatsompop N. (2018). Effects of DCP as a free radical producer and HPQM as a biocide on the mechanical properties and antibacterial performance of in situ compatibilized PBS/PLA blends. Polym. Test..

[45] Zhang B., Sun B., Bian X., Li G., Chen X. (2017). High Melt Strength and High Toughness PLLA/PBS Blends by Copolymerization and in Situ Reactive Compatibilization. Ind. Eng. Chem. Res..

[46] Li L., Song G., Tang G. (2013). Novel Biodegradable Polylactide/Poly(butylene succinate) Composites via Cross-Linking with Methylene Diphenyl Diisocyanate. Polym.-Plast. Technol. Eng..

[47] Chinda C., Hongsriphan N. Ductility Enhancement of Poly(butylene succinate)/Poly(lactic acid) Blend by the Use of Glycidyl Methacrylate. https://www.researchgate.net/publication/328213636_Ductility_enhancement_of_polybutylene_succinatepolylactic_acid_blend_by_the_use_of_glycidyl_methacrylate.

[48] Ji D., Liu Z., Lan X., Wu F., Xie B., Yang M. (2014). Morphology, rheology, crystallization behavior, and mechanical properties of poly(lactic acid)/poly(butylene succinate)/dicumyl peroxide reactive blends. J. Appl. Polym. Sci..

[49] Chen G.-X., Kim H.-S., Kim E.-S., Yoon J.-S. (2005). Compatibilization-like effect of reactive organoclay on the poly(l-lactide)/poly(butylene succinate) blends. Polymer.

[50] Manning S.C., Moore R.B. (1999). Reactive compatibilization of polypropylene and polymide-6,6 with carboxylated and maleated polypropylene. Polym. Eng. Sci..

[51] Galeski A., Bartczak Z., Martuscelli E., Martuscelli E., Musto P., Ragosta G. (1996). Nucleation process in thoughend plastics. Advanced Routes for Polymer Toughening.

[52] Kim Y.J., Park O.O. (1999). Miscibility and Biodegradability of Poly(Butylene Succinate)/Poly(Butylene Terephthalate) Blends. J. Polyers Environ..

[53] Luzi F., Fortunati E., Jiménez A., Puglia D., Pezzolla D., Gigliotti G., Kenny J.M., Chiralt A., Torre L. (2016). Production and characterization of PLA_PBS biodegradable blends reinforced with cellulose nanocrystals extracted from hemp fibres. Ind. Crop. Prod..

[54] Pivsa-Art W., Fujii K., Nomura K., Aso Y., Ohara H., Yamane H. (2016). Isothermal crystallization kinetics of talc-filled poly(lactic acid) and poly(butylene succinate) blends. J. Polym. Res..

[55] Xu H., Teng C., Yu M. (2006). Improvements of thermal property and crystallization behavior of PLLA based multiblock copolymer by forming stereocomplex with PDLA oligomer. Polymer.

[56] Jing Z., Shi X., Zhang G., Lei R. (2015). Investigation of poly(lactide) stereocomplexation between linear poly(L-lactide) and PDLA-PEG-PDLA tri-block copolymer. Polym. Int..

[57] Li Z., Tan B.H., Lin T., He C. (2016). Recent advances in stereocomplexation of enantiomeric PLA-based copolymers and applications. Prog. Polym. Sci..

[58] Zhang X., Meng L., Li G., Liang N., Zhang J., Zhu Z., Wang R. (2016). Effect of nucleating agents on the crystallization behavior and heat resistance of poly(l-lactide). J. Appl. Polym. Sci..

[59] Mallet B., Lamnawar K., Maazouz A. (2014). Improvement of blown film extrusion of poly(Lactic Acid): Structure-Processing-Properties relationships. Polym. Eng. Sci..

[60] Osborn K.R., Jenkins W.A. (1992). Plastic Films: Technology and Packaging Applications.

[61] (2018). Communication from the Commission to the European Parliament, the Council, the European Economic and Social Committee and the Committee of the Regions: A European Strategy for Plastics in a Cicular Economy.

[62] nova-Institute Biodegradable, Bio-Based Polymers in Various Environments. http://bio-based.eu/downloads/biodegradable-bio-based-polymers-in-various-environments/.

[63] Chinaglia S., Tosin M., Degli-Innocenti F. (2018). Biodegradation rate of biodegradable plastics at molecular level. Polym. Degrad. Stab..

[64] Mochizuki M., Hirami M. (1997). Structural Effects on the Biodegradation of Aliphatic Polyesters. Polym. Adv. Technol..

[65] La Mantia F.P., Morreale M., Botta L., Mistretta M.C., Ceraulo M., Scaffaro R. (2017). Degradation of polymer blends: A brief review. Polym. Degrad. Stab..

[66] Zhang S.J., Tang Y.W., Cheng L.H. (2013). Biodegradation Behavior of PLA/PBS Blends. AMR.

[67] Fleischer G., Habashi F., Menges G., Bilitewski B., Loll U. (2011). Waste, 5. Recycling. Ullmann’s Encyclopedia of Industrial Chemistry.

[68] Tsuneizumi Y., Kuwahara M., Okamoto K., Matsumura S. (2010). Chemical recycling of poly(lactic acid)-based polymer blends using environmentally benign catalysts. Polym. Degrad. Stab..

[69] Bioplasticsmagazine Bio-Based Polymers Have Potential in Biomedicine, Agricultural Markets. https://www.bioplasticsmagazine.com/en/news/meldungen/20150407-New-report-shows-promise-bioplastics.php.

[70] Soroudi A., Jakubowicz I. (2013). Recycling of bioplastics, their blends and biocomposites: A review. Eur. Polym. J..

[71] FKuR Kunststoff GmbH Bio-Flex S 5630 WH.

[72] nova-Institut GmbH (2010). K 2010: FKuR Präsentiert neue Biokunststoffe für Folien- und Spritzgießanwendungen. Bio-Flex Compounds Erweitern das Nachhaltige Produkt-Portfolio der Kunststoff GmbH.

